# Commercial Coffee Wastes as Materials for Adsorption of Heavy Metals from Aqueous Solutions

**DOI:** 10.3390/ma5101826

**Published:** 2012-10-10

**Authors:** George Z. Kyzas

**Affiliations:** Department of Petroleum and Natural Gas Technology, Technological Educational Institute of Kavala, Kavala GR-654 04, Greece; E-Mail: georgekyzas@gmail.com; Tel./Fax: +30-2310-858607.

**Keywords:** coffee wastes, heavy metals, Cu(II), Cr(VI), adsorption, low-cost materials

## Abstract

This work aims to study the removal of Cu(II) and Cr(VI) from aqueous solutions with commercial coffee wastes. Materials with no further treatment such as coffee residues from café may act as adsorbents for the removal of Cu(II) and Cr(VI). Equilibrium data were successfully fitted to the Langmuir, Freundlich and Langmuir-Freundlich model (L-F). The maximum adsorption capacity of the coffee residues can reach 70 mg/g for the removal of Cu(II) and 45 mg/g for Cr(VI). The kinetic data were fitted to pseudo-first, -second and -third order equations. The equilibrium was achieved in 120 min. Also, the effect of pH on adsorption and desorption was studied, as well as the influence of agitation rate. Ten cycles of adsorption-desorption were carried out revealing the strong reuse potential of these low-cost adsorbents; the latter was confirmed from a brief economic approach.

## 1. Introduction

Main heavy metals contamination of water is a serious threat to the globe ecosystem. Many industries such as metal plating, mining operation, and tanneries release wastewaters contaminated with heavy metals into the environment [1]. So, their removal from contaminated waters has become a major topic of research in recent years, due to the toxicological problems caused by the metal ions to the environment and to human health. Various processes of heavy metals elimination are used, such as precipitation, electro precipitation, electro coagulation, cementing and separation by membrane, the solvent extraction and the exchange of ions on resins [2]. However, these processes are not economical enough for wastewater treatment. Strict environmental protection legislation and public environmental concerns lead the global search for novel and low-cost techniques to remove heavy metals from industrial wastewater [1,2]. So, recent research is directed to developing cost-effective technologies for the removal of metal ions from aqueous solutions.

Adsorption is considered to be quite attractive in terms of its efficiency of removal from dilute solutions. Although the use of common materials (activated carbon [1], chitosan [3], zeolite, clay [4]) is still very popular due to the high adsorption capacity, they are expensive, too. Thus, there is a growing demand to find relatively efficient, low-cost and easily available adsorbents for the adsorption of heavy metls, particularly if the adsorbents are the wastes. Researchers were oriented towards no expensive adsorbents. However, there is a lack of literature dealing with the possible application of commercial coffee wastes as adsorbents. Under the title of “coffee wastes” are generally called the solid wastes discarded from the extraction process of instant coffee manufacturing, and the final residues originated from cafeterias.

In the last years, the instant coffee industry has experienced a constant growth as instant coffee has become one of the most popular kinds of coffee drunk by millions of people around the world. As a consequence, large amounts of coffee grounds, which are the solid residues obtained during the processing of coffee powder with hot water or steam to prepare instant coffee, have been generated worldwide (in the order of 6 million tons per year) [5].

Since sustainable development should be prioritized, the development of techniques for giving additional value and reusing this type of residues should be sought. In view of the aforementioned, the objective of this study was to investigate the feasibility of using untreated coffee residues from cafeterias directly as natural biosorbents for the removal of heavy metals (Cu(II) and Cr(VI)) from synthetic aqueous media. It is a great of interest to treat heavy metal effluents with untreated natural residues. Different parameters of adsorption process were studied, such as the effect of pH, contact time, agitation rate, ion initial concentration. Another very important factor studied was the reuse of the coffee residuals in sequential adsorption-desorption cycles. Also, an economic approach was realized to show: (i) the feasibility/effectiveness of the coffee grounds used as potential adsorbents; (ii) how the respective treated sample of coffee is greater and more economically feasible than the untreated sample. A useful perspective, after the batch scale modules on lab tests of the current work, will be the fixed beds in a possible next step, and later the adjustment in pilot scale tests.

## 2. Materials and Methods

### 2.1. Materials

The samples of coffee residues were collected after roasting a special variety of coffee drinks (“Greek coffee” drinks) and kindly donated by a local café (Thessaloniki, Greece). Also, CuSO_4_∙5H_2_O (Fluka, 98% purity) and K_2_Cr_2_O_7_ (Fluka, puriss. p.a. ≥ 99.0%) were used for the preparation of Cu(II) and Cr(VI) adsorbate stock solutions, respectively. All experiments were repeated 4 times and the experimental points (in adsorption/desorption figures) display the average of the data found. This was realized in order to diminish the experimental faults.

### 2.2. Types of Coffee Wastes

Two types of agricultural wastes were used in the current work: (i) Untreated Coffee Residues (abbreviated hereafter as UCR) from cafeterias and constitute a waste (without no further treatment, just only dried at the ambient air and then sieved); (ii) Treated Coffee Residues (abbreviated hereafter as TCR), which were washed with distilled water to remove dirt and color, and dried at 105 °C for 5 h in a convection oven. Then, they were then treated with 2% formaldehyde solution in order to reduce organic leaching and avoid mould formation during batch adsorption [4]. Both two types of residues were in powder form (475–525 μm) after sieving.

### 2.3. Adsorption Experiments

#### 2.3.1. Effect of pH

The effect of pH was conducted by mixing 1 g/L of adsorbent with 50 mL metal single-component solution (50 mg/L). The pH value, ranging between 2 and 12, was kept constant throughout the adsorption process by micro-additions of HNO_3_ (0.01 M) or NaOH (0.01 M). The suspension was shaken for 24 h (agitation rate = 140 rpm) into a water bath to control the temperature at 25 °C (Julabo SW-21C). The optimum pH selected was pH = 5 both for Cu(II) and Cr(VI).

#### 2.3.2. Effect of Contact Time

Kinetic experiments were performed by mixing 1 g/L of sorbent with 50 mL metal single-component solution (50 mg/L). The suspensions were shaken for 24 h at pH = 5 (both for Cu(II) and Cr(VI)) in water bath at 25 °C (agitation rate = 140 rpm). Samples were collected at fixed intervals (5–30 min, 1–24 h). The following three equations (pseudo-first order, Equation 1; pseudo-second order, Equation 2; pseudo-third order, Equation 3) were selected to fit the experimental kinetic data [6]:

(1) $$C_{t}=C_{0}-(C_{0}-C_{e})\left(1-e^{-k_{1}t}\right)$$

(2) $$C_{t}=C_{0}-(C_{0}-C_{e})\left(1-\frac{1}{1+k_{2}t}\right)$$

(3) $$C_{t}=C_{0}-(C_{0}-C_{e})\left(1-\frac{1}{{\left(1+2k_{3}t\right)}^{1/2}}\right)$$

where, k_1_, k_2_, k_3_ (min^−1^) are the rate constants for the pseudo-first, -second and -third order adsorption model and C_0_, C_t_, C_e_ (mg/L) are the initial, transient and equilibrium concentrations of metal in the aqueous solution, respectively.

#### 2.3.3. Effect of Initial Metal Concentration—Isotherms

The effect of initial metal (single-component) concentration on equilibrium was realized by mixing 1 g/L of both samples (UCR, TCR) of adsorbents with 50 mL of metal solutions of different initial concentrations (0–150 mg/L). The suspensions were shaken for 24 h at pH = 5 (both for Cu(II) and Cr(VI)) in water bath at 25 °C (agitation rate = 140 rpm).

The resulted equilibrium data were fitted to the Langmuir (Equation 4) [7], Freundlich (Equation 5) [8] and Langmuir-Freundlich (L-F) (Equation 6) isotherm model [9], expressed by the following respective equations:

(4) $$Q_{e}=\frac{Q_{\text{max}}K_{L}C_{e}}{1+K_{L}C_{e}}$$

(5) $$Q_{e}=K_{F}{C_{e}}^{1/n}$$

(6) $$Q_{e}=\frac{Q_{\text{max}}{\left(K_{\text{LF}}C_{e}\right)}^{b}}{1+{\left(K_{\text{LF}}C_{e}\right)}^{b}}$$

where, Q_e_ (mg/g) is the equilibrium metal concentration in the solid phase; Q_max_ (mg/g) is the maximum amount of sorption; K_L_ (L/mg) is the Langmuir sorption equilibrium constant; K_F_ (mg^1−1/n^ L^1/n^/g) is the Freundlich constant representing the sorption capacity, n is the constant depicting the sorption intensity, K_LF_ ((L/mg)^1/b^) is the Langmuir-Freundlich constant, and b (dimensionless) is the L-F heterogeneity constant.

The amount of total metal uptake at equilibrium Q_e_ (mg/g) was calculated using the mass balance equation (Equation 7):

(7) $$Q_{e}=\frac{(C_{0}-C_{e})V}{m}$$

where, m (g) is the mass of adsorbent; V (L) the volume of adsorbate; C_0_ and C_e_ (mg/L) are the initial and equilibrium metal concentrations (single-component) in the liquid phase, respectively.

#### 2.3.4. Effect of Agitation Rate

The effect of agitation rate on equilibrium was observed by mixing 1 g/L of both samples (UCR, TCR) of adsorbents with 50 mL of 50 mg/L metal solutions (single-component). The suspensions were shaken for 24 h at pH = 5 (both for Cu(II) and Cr(VI)) in water bath at 25 °C. The rate of agitation was ranged from 60 to 180 rpm.

### 2.4. Desorption and Reuse Experiments

After the adsorption experiments (where the coffee residues were exposed to 50 mg/L of metal single-component solution at 25 °C (at pH = 5 both for Cu(II) and Cr(VI)), the samples were collected and filtered, using fixed pore-sized membranes. A small fraction of the metals (1%–2%) and the adsorbent (1%) were retained on the filter membrane; these small variations due to filtration were neglected. Desorption experiments were realized by mixing the collected, after adsorption, amount of metal-loaded coffee residues (0.05 g) with aqueous solutions of 50 mL (same volume as in the adsorption step) over a pH range between 2 and 12, at 25 °C for 24 h (agitation rate = 140 rpm). This procedure was realized to determine the optimum desorption pH value of the metal-loaded adsorbents. To determine the reusability of the coffee residues, 10 sequential adsorption-desorption cycles were repeated, using the same adsorbents and following the experimental procedures described above in the optimum conditions found.

### 2.5. Analysis

Samples of the Cr(VI) solution were collected at pre-determined time intervals, filtered and analyzed using a UV-Vis Spectrophotometer (model U-2000, Hitachi), at λ_max_ = 540 nm, according to the 1,5-diphenyl-carbazide method. The respective samples of the solution of Cu(II) were analyzed by atomic absorption spectroscopy, using an Atomic Absorption Spectrophotometer (Perkin-Elmer AAnalyst 400) composed of FIAS 100 Flow Injection System. Due to the sensitivity of the instrument, the samples with high residual ion concentrations were diluted before the measurement. The aforementioned dilution was taken into account for all the calculations.

## 3. Results and Discussion

### 3.1. Effect of pH on Adsorption—Characterization

**Figure 1:**
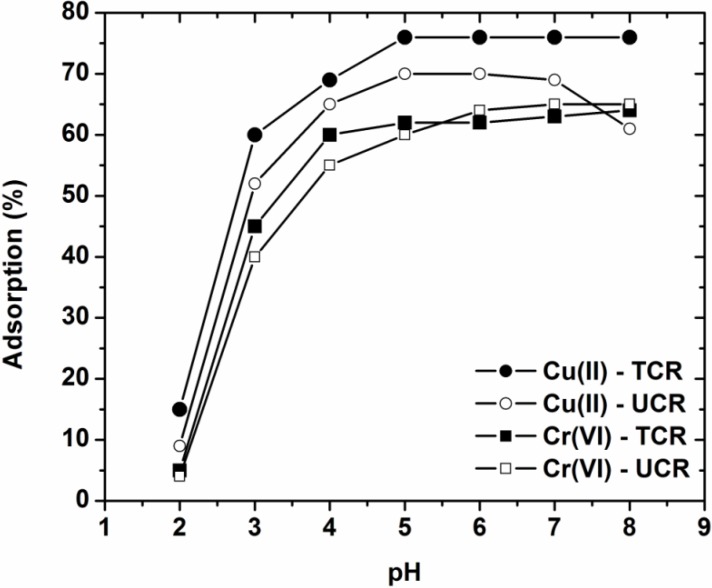
Effect of pH on adsorption of Cu(II) and Cr(VI) onto treated and untreated coffee residues (pH = 2–8, 50 mg/L ion concentration, 1 g/L adsorbent, T = 25 °C, 140 rpm, 24 h contact).

The effect of pH on the adsorption of Cu(II) and Cr(VI) onto the coffee residues (UCR, TCR) is depicted in Figure 1. It can be seen that the adsorption percentages are very low at strong acidic medium both for the treated adsorbent (TCR) and the untreated one (UCR).

At pH = 2, the removal percentages were 9% and 15% in the case of Cu(II) for adsorption onto UCR and TCR, respectively, and 4% and 5% in the case of Cu(II) for UCR and TCR, respectively. After pH 3 (Cu(II): 52%, UCR; 60%, TCR and Cr(VI): 40%, UCR; 45%, TCR), uptakes increase sharply up to pH = 5 (Cu(II): 70%, UCR; 76%, TCR and Cr(VI): 60%, UCR; 62%, TCR), and thereafter they stay almost constant for higher pHs. No pH values over 8 were studied since precipitation of heavy metals dominates. In all cases, the adsorption of ions onto the treated coffee residues (TCR) presented higher metal removal percentages. This can be explained taking in consideration that the impurities in residues compete and hinder the metal adsorption.

Another point of interest of these low-cost adsorbents is the non-easily explained adsorption mechanism. In the case of cations (as copper), the possible hypotheses are clear enough. The coffee materials primarily contain weak acidic and basic functional groups. It follows from the theory of acid-base equilibria that at 2 < pH < 7 the binding of heavy metals cations is determined primarily by the state of dissociation of the weak acidic groups. Carboxyl groups (–COOH) are the important (but not the unique) groups for metal uptake by these materials. At pH = 7, there are lower numbers of competing hydrogen ions and more in number ligands are exposed with negative charges, resulting in greater copper adsorption. The minimal adsorption at low pH may be due to the higher concentration and high mobility of H^+^, which are preferentially adsorbed rather than metal ions [10,11]. Increasing the pH of the solution, the lower number of H^+^ (with higher negative surface charge) results in more copper adsorption. In the case of anions (as Cr(VI)), the phenomena occurs during adsorption are not clear enough having many complexes and misunderstandings. Other researchers found an increase in adsorption increasing the pH of the solution [12], while other presented the opposite phenomenon [3]. This difference is mainly attributed to the various surface charge of each material. The co-existence of both carboxylic and basic groups on the surface of coffee wastes with nearly equal values, as it can be seen from characterization (see below), may explain the adsorption behavior of materials when the metal is anionic.

For both two heavy metals the optimum pH selected for further adsorption experiments (kinetics, isotherms etc) was 5. This was exported by considering and adopting some limitations, as: (i) copper introduces a limiting pH value of 5, above which its precipitation begins in the form of insoluble hydroxide [13,14]; (ii) the hydrolysis of Cr(VI) will predominantly produce anionic species at low pH values [13], thus, requiring a predominant positively charged surface for its efficient removal; (iii) on the idea of working in a condition close to that naturally established by the medium, thus, not requiring significant modifications. Other researches, who have studied the effect of pH on metal removal with coffee residues, studied the adsorption phenomenon in the optimum pH range 4–5, too [15,16,17]. The characterization will be important to further examine the properties of the adsorbents used.

So, characterization was realized to evaluate the surface chemistry and morphology of the low-cost materials used. The point of zero charge (PZC) was evaluated according to titration procedures described in literature [16,17,18]. Three aqueous solutions of different pH values (3, 6 and 11) were prepared. Several amounts of coffee wastes were added to 20 mL of each solution to prepare 0.05%, 0.1%, 0.5%, 1.0%, 3.0%, 7.0% and 10.0% w/w coffee/solutions. The aqueous suspensions containing different amounts of the adsorbent were let to equilibrate for 24 h under agitation (140 rpm) at 25 °C. The pH of each solution was then measured. The PZC was determined as the converging pH value from the pH *versus* adsorbent mass curve.

The determination of surface functional groups was based on the Boehm titration method [19]. Aqueous solutions of NaHCO_3_ (0.10 mol/L), Na_2_CO_3_ (0.05 mol/L), NaOH (0.10 mol/L) and HCl (0.10 mol/L) were prepared. 50 mL of these solutions were added to vials containing 1 g of dry coffee samples, let to be shaken (140 rpm) until equilibrium (24 h), and then filtered. Five solution blanks (without the adsorbent) were also prepared. In this way, the number of the basic sites was calculated from the amount of HCl that reacted with the coffee adsorbents. The various free acidic groups were derived using the assumption that NaOH neutralizes carboxyl, lactone and phenolic groups, Na_2_CO_3_ neutralizes carboxyl and lactone and NaHCO_3_ neutralizes only carboxyl groups. The excess of base or acid was then determined by back titration using NaOH (0.10 mol/L) and HCl (0.10 mol/L) solutions [18].

Scanning electron microscopy (SEM) observations of the prepared particles were carried out using a JEOL JMS-840A scanning microscope equipped with an energy-dispersive X-ray (EDX) Oxford ISIS 300 micro-analytical system. All of these surfaces were coated with a thin layer of carbon black to avoid charging under the electron beam. The surface area was also determined with a Micromeritics BET (Brunauer, Emmett and Teller) surface area analyzer, model TriStar 3000, by means of adsorption of ultra pure nitrogen.

The composition of coffee residues was determined as 44% water, 12% protein, 14% lipids, 25% carbohydrates and 5% ash. According to Boehm method [19], the functional groups at the surface of adsorbents were carboxylic 0.97 mmol/g_COF_ and basic 0.93 mmol/g_COF_, followed by phenolic 0.14 mmol/g_COF_ and 0.11 lactonic mmol/g_COF_.

**Figure 2:**
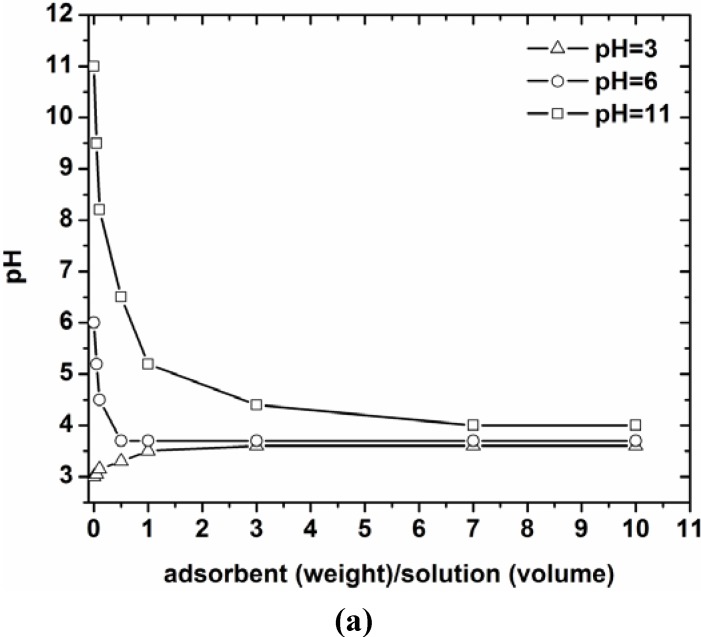

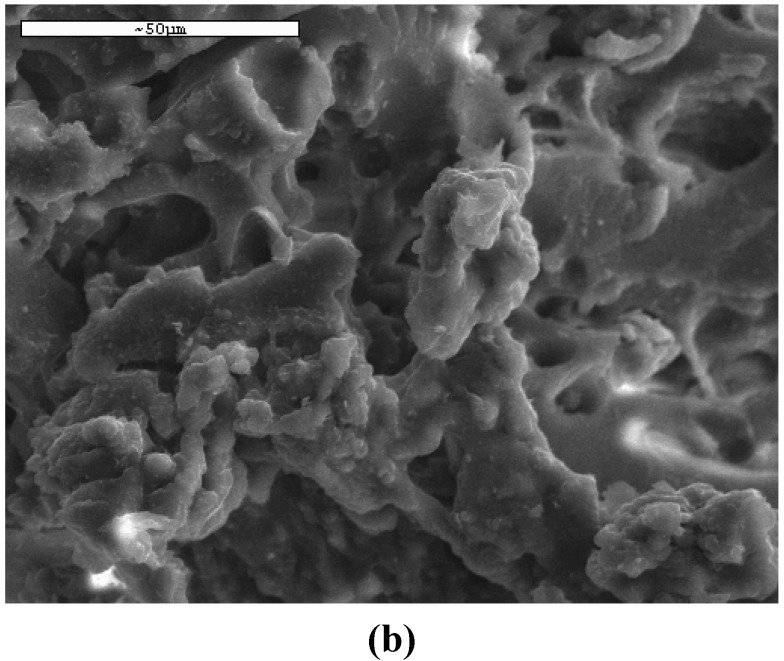
(**a**) Determination of pH_PZC_ for the coffee residues; (**b**) Scanning Electron Micrograph of coffee residues.

The energy dispersive X-ray microanalysis (SEM/EDX) of the coffee wastes indicates mainly the presence of oxygen (64.11%) and carbon (30.59%). Also a variety of other elements (5.30%) was also determined mainly as admixtures (K, Na, Mg, P, S). The experimental titration curves for PZC determination are presented in Figure 2a. These results indicated the PZC values in the range of 3.3–3.5. So, for pH values above of 3.4, a predominant negatively charged surface of adsorbents is occurred. At lower pH values, the surface charge may get mainly positively charged. The aforementioned consideration was proved and extensively explained in line with the adsorption mechanism of dyes onto coffee surface in previously published work [16,17].

Also, Figure 2b presents the morphology of COF according to SEM micrographs taken. It is obvious that its surface was not smooth, but full of cavities. These cavities can be characterized as channels onto the surface of adsorbents instead of pores, given the small surface area calculated from BET analysis (~2.3 m^2^/g).

### 3.2. Effect of Contact Time

**Figure 3:**
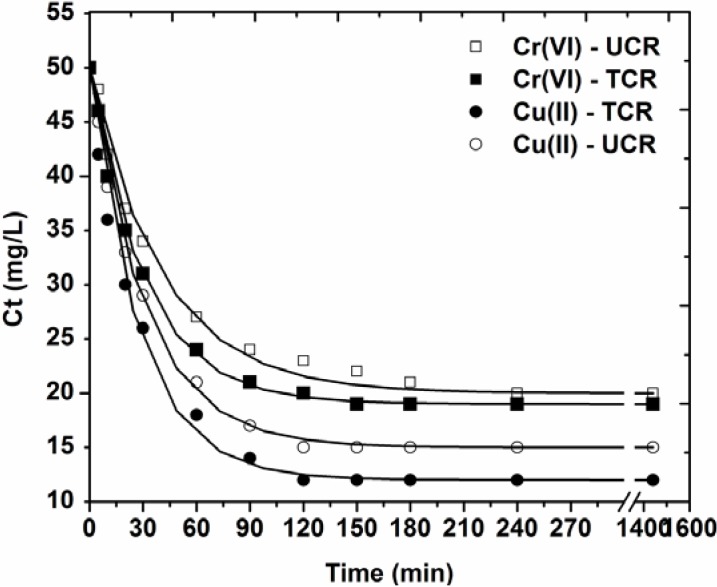
Effect of contact time on adsorption of Cu(II) and Cr(VI) onto treated and untreated coffee residues (pH = 5, 50 mg/L ion concentration, 1 g/L adsorbent, T = 25 °C, 140 rpm, 5 min–24 h contact).

Figure 3 depicts the effect of contact time on the adsorption behavior of the low-cost materials used.

The plots could be split in three distinct regions: (i) 0–20 min, which indicates the instantaneous adsorption of ions, suggesting rapid external diffusion and surface adsorption; (ii) 2–180 min, shows a gradual equilibrium; (iii) 3–24 h, indicates the equilibrium state [20]. However, it is obvious that the adsorbate-adsorbent system of Cu(II)-coffee reached in equilibrium faster (~120 min), while the respective time for Cr(VI) was 180 min. This could be attributed to the more favor conditions at the pH = 5 studied, because in these conditions the relatively negatively surface of coffee adsorbent attracts easily the positively charged copper ions.

Table 1 presents the kinetic parameters resulted by fitting the pseudo-first, -second and -third order equation to the experimental data. According to the correlation coefficients (R^2^) exported, the best fitting was for the pseudo-first order equation (0.994 < R^2^ < 0.998), while the pseudo-second (0.963 < R^2^ < 0.972) and pseudo-third (0.872 < R^2^ < 0.889) order equations presented enough lower coefficients.

**Table 1:** Kinetic constants for the adsorption of Cu(II) and Cr(VI) onto coffee residues (UCR, TCR).

Adsorbate-Adsorbent	Pseudo-first order		Pseudo-second order		Pseudo-third order	
	k_1_	R^2^	k_2_	R^2^	k_3_	R^2^
	min^−1^	–	min^−1^	–	min^−1^	–
Cu(II)-UCR	0.032	0.998	0.061	0.963	0.128	0.872
Cu(II)-TCR	0.037	0.995	0.070	0.972	0.153	0.887
Cr(VI)-UCR	0.025	0.994	0.046	0.973	0.093	0.889
Cr(VI)-TCR	0.032	0.998	0.061	0.966	0.127	0.876

Apart from a modeling tool, the effect of contact time (kinetics) has an important physical meaning, which is to examine the functional time of a continuous adsorption-desorption system. In the current study, the adsorptive equilibrium was reached at 3 h. Making an hypothesis that the desorptive equilibrium was also reached at 3 h, it is obvious that one reuse cycle (adsorption-desorption) lasts 6 h. However, if another system reaches in adsorptive and desorptive equilibrium at *i.e.*, 6 h, the functional/use time period is changed. In a 24 h-continuous cycle of adsorption-desorption process, the first system is complete at 6 h, so 4 reuse cycles can be performed during 24 h, while in the other system, 2 reuse cycles can only be performed. This can save functional/use time, money, energy *etc.*

### 3.3. Effect of Initial Metal Concentration

The fitting parameters of the three models are listed in Table 2. The values of correlation coefficient (R^2^ ~ 0.998), which is an indication of the fitting success, confirm that the L-F model results in closer prediction of the isotherm compared with the experimental data.

**Table 2:** Equilibrium parameters for the adsorption of Cu(II) and Cr(VI) onto coffee residues (UCR, TCR).

Ion-Adsorbent	Langmuir equation			Freundlich equation			L-F equation			
	Q_max_	K_L_	R^2^	K_F_	n	R^2^	Q_max_	K_LF_	b	R^2^
	mg/g	L/mg	–	mg^1−1/n^ L^1/n^ g^−1^	–	–	mg/g	(L/mg)^1/b^	–	–
Cu(II)-UCR	49.34	0.222	0.978	16.842	4.02	0.964	59.18	0.132	0.637	0.996
Cu(II)-TCR	56.90	0.229	0.973	19.245	3.94	0.924	69.97	0.125	0.612	0.993
Cr(VI)-UCR	38.68	0.139	0.995	11.739	3.90	0.915	37.03	0.150	1.180	0.998
Cr(VI)-TCR	43.75	0.136	0.996	12.505	3.67	0.940	44.82	0.129	0.932	0.996

**Figure 4:**
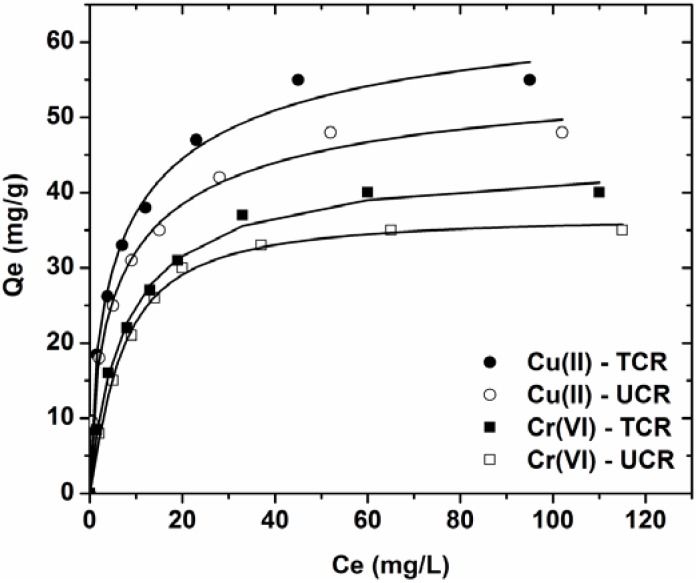
Effect of initial ion concentration on adsorption of Cu(II) and Cr(VI) onto treated and untreated coffee residues (pH = 5, 0–50 mg/L ion concentration, 1 g/L adsorbent, T = 25 °C, 140 rpm, 5 min–24 h contact).

The effect of initial metal concentration on the adsorption metal loading of the coffee residues is graphically given by Figure 4. Typical adsorption isotherms were observed for both powder and beads materials. Data showed an increase in the amount of metal adsorbed onto the coffee adsorbents, when the initial metal concentration was increased.

An important experimental finding is that the change of the maximum adsorption capacity (Q_max_) between the treated and untreated material was approximately only 10 mg/g. (Cu(II): 5 mg/g, UCR; 69 mg/g, TCR and Cr(VI): 37 mg/g, UCR; 44 mg/g, TCR). This can be useful as will be described in Section 3.6. about the perspectives.

### 3.4. Effect of Agitation Rate

Agitation is an important parameter in adsorption phenomena, influencing the distribution of the solute in the bulk solution and the formation of the external boundary film. Generally, the rate of ion removal is influenced by the rate of agitation and the uptake increased with stirring rate. The rate of agitation reduced the boundary-layer resistance and increased the mobility of the system. The increase of agitation lowers the external mass transfer effect.

**Figure 5:**
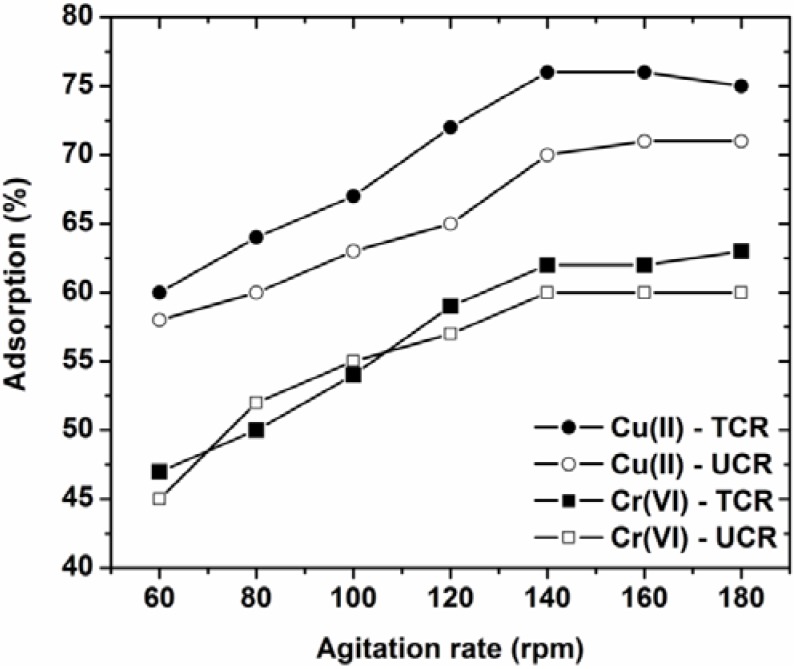
Effect of agitation rate on adsorption of Cu(II) and Cr(VI) onto treated and untreated coffee residues (pH = 5, 50 mg/L ion concentration, 1 g/L adsorbent, T = 25 °C, 60–180 rpm, 24 h contact).

The effect of agitation rate on metal adsorption is presented in Figure 5. The trend of agitation curve is similar for all the adsorbate-adsorbent systems studied: increasing the agitation speed, a slight increase in the metal removal occurs.

However, over 140 rpm, there is no any increase. For low agitation speed 60–80 rpm, the metal ions can hard “find” the possible active sites on the coffee residues. With the enhancement of the agitation speed, the potential adsorption increases, reaching a rate-limit over which there is no significant change. So, the optimum agitation rate determined was 140 rpm.

### 3.5. Desorption and Reuse

**Figure 6:**
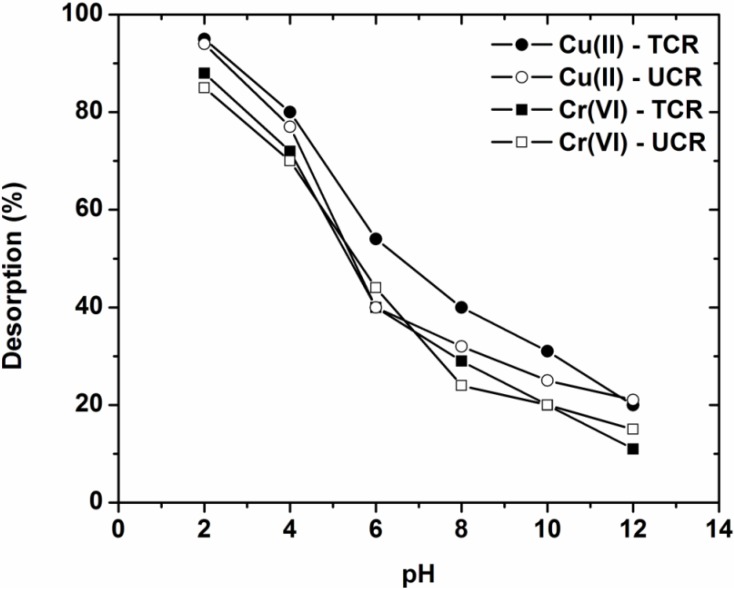
Effect of pH on desorption of Cu(II) and Cr(VI) onto treated and untreated coffee residues (pH = 2–12, 1 g/L adsorbent, T = 25 °C, 140 rpm, 24 h contact).

A further goal of any adsorbent used is the reuse potential. So, before the investigation of the reuse, desorption experiments were carried out to find the optimum pH-desorption conditions. Figure 6 shows the effect of pH on desorption of heavy metals studied form the low-cost materials used.

Both for Cu(II) and Cr(VI) the strong acidic conditions favor the desorption of metals at high percentages (Cu(II)-UCR, 94%; Cr(VI)-UCR, 85%). In contrast, in alkaline conditions the desorption is taking place in low percentages (Cu(II)-UCR, 21%; Cr(VI)-UCR, 15%). So, the pH value selected for the further reuse experiments (adsorption-desorption cycles) was pH = 2.

**Figure 7:**
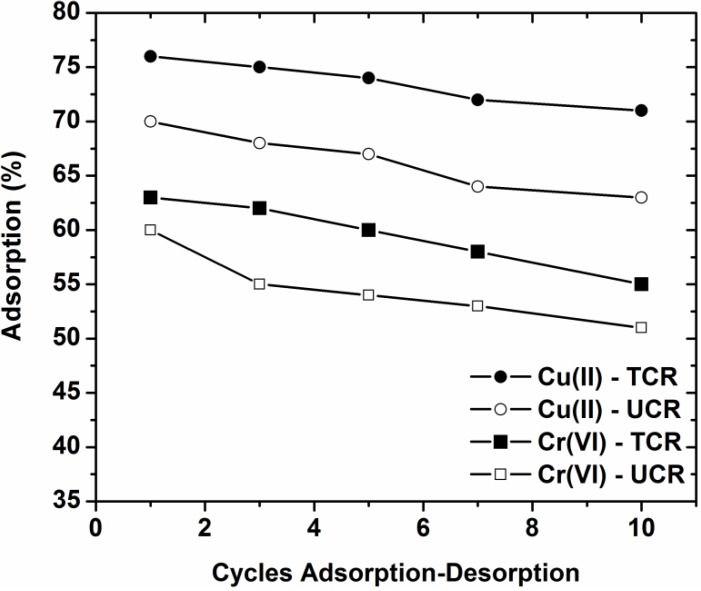
Cycles of adsorption-desorption for the reuse of untreated coffee as adsorbents for Cu(II) and Cr(VI) removal.

To investigate the possibility of reuse of the low-cost materials of the current study, sequential adsorption-desorption experiments in batch mode were conducted for ten cycles. Figure 7 shows that the reduction in adsorption percentages from the 1st to 10th cycle was 7% for Cu(II)-UCR, 6% for Cu(II)-TCR, 10% for Cr(VI)-UCR and 9% for Cr(VI)-TCR.

The decrease of the adsorption efficiency occurred can be attributed to several reasons as: (i) a progressive saturation of the active sites/groups of the adsorbent by metal ions; (ii) a degradation of material due to extreme pH conditions. In addition, a progressive blocking of the active sites of the adsorbent by impurities in the case of untreated coffee residues caused a slight decrease in the adsorption potential compared to the treated ones. In the cases of low-cost materials used as adsorbents and especially for copper and chromium removal with coffee residues, the loss of their adsorptive ability is characterized as enough low, very sufficient for further repeated use [1].

### 3.6. Economic Perspectives

The coffee residues used in the study is the result after the roasting of a special variety of coffee (Greek coffee). To have a more comparative view, apart from the untreated form (just dry), a slight modification was realized to obtain a more pure form. However, according to the experimental data, the changes in the crucial factors of adsorption (Q_max_, adsorption) and reuse (cycles) were only slight, and easily can be neglected. Also, the regeneration step of these adsorbents is easy. They can be regenerated by desorption at low cost if required. They are easily regenerated by a washing solvent since the interaction between the pollutant and adsorbent is driven mainly by electrostatic, hydrophobic and ion-exchange interactions. The desorption side of the process gives the pollutant in a concentrated form and restores the material close to the original condition for effective reuse with no physical-chemical changes or damage. The regeneration of saturated carbon by thermal and chemical procedure is known to be expensive, and results in loss of the adsorbent. The adsorption properties are also reproducible. After saturation, the adsorption capacity value remains unchanged.

Practically and numerically, there are coffee residues with no further use in nutrition cycle (coffee) which can be used and re-used up to 10 times adsorbing approximately 70 mg of Cu(II) and 40 mg of Cr(VI) per gram. This is directly comparative with other examples of coffee residues, where the cost of preparation was higher. According to Baek [21], the comparison between the cost of the use of activated carbon and low-cost materials is up to 15 times larger. Given a gross estimation of the economic superiority is the cost of use of the two common materials for metal adsorption; Activated Charcoal Norit ROX 125.5 €/kg and chitosan 7–28 €/kg [22]. Taking into consideration that the coffee residues of the current study has nearly zero cost (for the untreated form), there is a great potential to further and continuous use in bed columns or possible pilot scale.

## 4. Conclusions

In this study, two types of coffee residues (treated and untreated) were used as low-cost adsorbents for the removal of Cu(II) and Cr(VI) from aqueous solutions. The experimental observations are summarized below:

• The pH selected as optimum for further adsorption experiments was pH = 5, where the adsorbents presented the maximum removal just before the pH-zone of 5–8 where precipitation and hydrolysis phenomena dominate. The surface charge of the adsorbents was confirmed through the determination of PZC for each material.
• Equilibrium data were fitted to the Lanmguir, Freundlich and Langmuir-Freundlich (L-F) model. The best correlation was for L-F model (R^2^ ~ 0.998).
• Kinetic data were fitted to the pseudo-first, -second and -third order model. The best correlation was for pseudo-first order equation (R^2^ ~ 0.996).
• The optimum agitation rate for the current adsorption phenomenon was determined to be 140 rpm.
• The optimum pH found after desorption experiments was pH = 2 both for Cu(II) and Cr(VI).
• After 10 cycles of adsorption-desorption, the reduction in adsorption percentages from the 1st to 10th cycle was approximately 7% for both coffee residues and ions.
